# Nutrient Limitation Causes Differential Expression of Transport- and Metabolism Genes in the Compartmentalized Anammox Bacterium *Kuenenia stuttgartiensis*

**DOI:** 10.3389/fmicb.2020.01959

**Published:** 2020-08-13

**Authors:** Marjan J. Smeulders, Stijn H. Peeters, Theo van Alen, Daan de Bruijckere, Guylaine H. L. Nuijten, Huub J. M. op den Camp, Mike S. M. Jetten, Laura van Niftrik

**Affiliations:** Department of Microbiology, Institute for Water and Wetland Research, Faculty of Science, Radboud University Nijmegen, Nijmegen, Netherlands

**Keywords:** anammox, planctomycete, anammoxosome, amtB, focA, narK, transcriptomics, nutrient limitation

## Abstract

Anaerobic ammonium-oxidizing (anammox) bacteria, members of the “*Candidatus* Brocadiaceae” family, play an important role in the nitrogen cycle and are estimated to be responsible for about half of the oceanic nitrogen loss to the atmosphere. Anammox bacteria combine ammonium with nitrite and produce dinitrogen gas via the intermediates nitric oxide and hydrazine (anammox reaction) while nitrate is formed as a by-product. These reactions take place in a specialized, membrane-enclosed compartment called the anammoxosome. Therefore, the substrates ammonium, nitrite and product nitrate have to cross the outer-, cytoplasmic-, and anammoxosome membranes to enter or exit the anammoxosome. The genomes of all anammox species harbor multiple copies of ammonium-, nitrite-, and nitrate transporter genes. Here we investigated how the distinct genes for ammonium-, nitrite-, and nitrate- transport were expressed during substrate limitation in membrane bioreactors. Transcriptome analysis of *Kuenenia stuttgartiensis* planktonic cells showed that four of the seven ammonium transporter homologs and two of the nine nitrite transporter homologs were significantly upregulated during ammonium-limited growth, while another ammonium transporter- and four nitrite transporter homologs were upregulated in nitrite limited growth conditions. The two nitrate transporters were expressed to similar levels in both conditions. In addition, genes encoding enzymes involved in the anammox reaction were differentially expressed, with those using nitrite as a substrate being upregulated under nitrite limited growth and those using ammonium as a substrate being upregulated during ammonium limitation. Taken together, these results give a first insight in the potential role of the multiple nutrient transporters in regulating transport of substrates and products in and out of the compartmentalized anammox cell.

## Introduction

Anaerobic ammonium-oxidizing (anammox) bacteria play a major role in the biological nitrogen cycle where they contribute significantly to oceanic nitrogen loss and are estimated to be responsible for up to 50% of the dinitrogen gas release to the atmosphere (Francis et al., 2007; Lam and Kuypers, 2011). In addition, they are applied in wastewater treatment for cost-effective and environment-friendly nitrogen removal (Kartal et al., 2010; Lackner et al., 2014). Anammox bacteria belong to the family *Brocadiaceae* and order *Brocadiales* and so far five different genera have been described: “*Ca.* Kuenenia,” “*Ca.* Brocadia,” “*Ca.* Anammoxoglobus,” “*Ca.* Jettenia,” and “*Ca.* Scalindua” (Jetten et al., 2009). Anammox bacteria are not genetically tractable and grow slowly, with reported doubling times ranging from days to weeks (Lotti et al., 2015; Zhang and Okabe, 2020). They are cultivated as enrichment cultures of up to 95% purity in bioreactor systems. Although anammox cells grow generally in larger cell aggregates, the “type strain” *Kuenenia stuttgartiensis* and *Brocadia sinica* can be cultivated as a planktonic enrichment culture (van der Star et al., 2008; Zhang and Okabe, 2017).

Anammox bacteria have a unique cell organelle, the anammoxosome, which occupies a major part of the cell volume (van Niftrik and Jetten, 2012). The anammox reaction takes place in the anammoxosome and is postulated to be coupled to energy conservation over the anammoxosome membrane (van Niftrik et al., 2008a, b; Neumann et al., 2014). The anammox reaction proceeds via three consecutive redox reactions (for a review see Kartal and Keltjens, 2016). First, a nitrite reductase performs the one-electron reduction of nitrite to nitric oxide. It is proposed that this step is carried out by distinct enzymes in different anammox species, more specifically the iron cytochrome cd1 NirS (*K. stuttgartiensis* and marine *Scalindua species*), the octaheme hydroxylamine oxidoreductase kustc0458 (*K. stuttgartiensis* RU1), or the copper containing NirK (*Jettenia caeni*) (Strous et al., 2006; Kartal et al., 2011b, 2013; Hira et al., 2012; van de Vossenberg et al., 2013; de Almeida et al., 2016; Speth et al., 2016; Hu et al., 2019).

The next step in the anammox reaction is the condensation of nitric oxide and ammonium with four electrons to produce hydrazine performed by the hydrazine synthase (HZS) (Dietl et al., 2015). Finally, hydrazine is oxidized to dinitrogen gas by the hydrazine dehydrogenase (HDH) (Maalcke et al., 2016; Akram et al., 2019a). The oxidation of hydrazine releases four low potential electrons that are proposed to be shuttled via soluble cytochromes to an electron transport chain in the anammoxosome membrane and ultimately back to the nitrite reductase and hydrazine synthase (Strous et al., 2006; Kartal et al., 2013; Kartal and Keltjens, 2016). The electrons would flow via an electron transport chain consisting of Rieske-heme *b* complexes (*bc*1 complexes) and a quinone pool in order to establish a proton motive force (and subsequent ATP synthesis) across the anammoxosome membrane. Some electrons are “lost” from the cycle to provide the reducing power for carbon fixation and assimilation, and these lost electrons are thought to be supplemented by the oxidation of nitrite to nitrate by the nitrite oxidoreductase Nxr. This produces the nitrate that is observed as an additional product when anammox bacteria are grown on nitrite and ammonium (van de Graaf et al., 1997; Kartal and Keltjens, 2016).

As the anammox reaction takes place in the anammoxosome, the substrates and products need to cross both the outer- and cytoplasmic membrane as well as the anammoxosome membrane. In other prokaryotes, ammonium-, nitrite- and nitrate transport is facilitated by dedicated transporter proteins that reside in the cytoplasmic membrane. Ammonium transport is mediated by the AmtB/Mep/Rh family of proteins that are found in all domains of life (Andrade and Einsle, 2007; McDonald and Ward, 2016). The ammonium transporter consists of a homotrimer and each monomer contains an ammonium channel (Zheng et al., 2004). In different members of the AmtB/Mep/Rh family, the substrate for transport may vary: NH_3_ for Rh family transporters and NH_4_^+^ or H^+^/NH_3_ for Amt and Mep transporters (Neuhauser et al., 2014; Wacker et al., 2014; Baday et al., 2015). GlnK P_*II*_ regulators modulate ammonium transporters by binding near the exit port and thereby blocking the transport channel when ammonium is present in high levels (Conroy et al., 2007; Gruswitz et al., 2007). These GlnK P_*II*_ regulators and AmtB transporters are conserved gene pairs in prokaryotes (Thomas et al., 2000; Coutts et al., 2002).

Nitrite transport is mediated via the formate/nitrite transporter (FNT) family, containing FocA, NirC and HSC transporters that are associated with formate, nitrite and hydrosulphide transport, respectively (Lu et al., 2013; Mukherjee et al., 2017). They all transport anions and different weak acids and transport is driven via proton binding, resulting in transfer of neutral substrate through the pore, similar to the mechanism proposed for AmtB transporters (Atkovska and Hub, 2017; Wiechert and Beitz, 2017a, b). Nitrate is transported via the NarK family transporters which are members of the Major Facilitator Superfamily (MFS) of transporters (Andrade and Einsle, 2013; Noinaj and Buchanan, 2014). Two NarK subtypes have been identified: the NarK1 type transporters are associated with nitrate assimilation and transport is thought to occur via nitrate/proton symport. The NarK2 type transporters are generally used during dissimilatory nitrate reduction and are thought to act as nitrate:nitrite antiporters. This would give them a double function of nitrate import and removal of the toxic nitrite produced by nitrate reduction (see Goddard et al., 2017 for review).

All published anammox (meta)genomes harbor multiple copies of ammonium, nitrite and nitrate transporters that could facilitate transport into- or out of the anammoxosome crossing several membrane bilayers (Strous et al., 2006; Speth et al., 2012; van de Vossenberg et al., 2013; Oshiki et al., 2015; Speth et al., 2015; Frank et al., 2018). The closed genome of the type strain *Kuenenia stuttgartiensis* MBR1 (Frank et al., 2018) encodes no less than seven AmtB-type ammonium transporter homologs, four P_*II*_ ammonium transport regulators (GlnK) and seven FNT-type transporters that are proposed to transport nitrite. There is also one annotated NirC-type nitrite transporter (Table 1). To transport nitrate out of the cell, two *narK* genes encoding nitrate transporters have been identified, both of which are of the NarK2-type and annotated as nitrate:nitrite transporters (Frank et al., 2018). In anammox bacteria, these may usually function in the opposite direction compared with other bacteria: exporting the by-product nitrate while simultaneously importing some additional nitrite as substrate for the anammox reaction. However the direction of transport will depend on the concentration gradients of nitrate and nitrite: Li et al. (2016) found that addition of nitrate to nitrite-stressed *Brocadia* cells alleviated nitrite toxicity, presumably via nitrate uptake coupled to nitrite export from the cell (Li et al., 2016).

**TABLE 1:** Nutrient transporter genes in the anammox bacterium *K. stuttgartiensis*.

Transporter	Prokka annotation*	Gene in *K. stuttgartiensis* MBR1*	Uniprot entry id	Homolog in *K. stuttgartiensis* RU1**
NH_4_^+^	amtB/amt1	KSMBR1_2086-conA	Q1PV65	kustc0381-xyl
	amt_1	KSMBR1_2627	Q1PX09	kustc1009
	amt_2	KSMBR1_2630	Q1PX06	kustc1012
	amt_3	KSMBR1_2632	Q1PX03	kustc1015
	amtb_2	KSMBR1_3866-HK	A0A2C9CK98	kuste3690-HK
	amtb_1	KSMBR1_3722-HK	A0A2C9CKL7	RU1_015c-HK***
	amt_4	KSMBR1_3716	A0A2C9CJU7	RU1_034***
P_*II*_ regulator	nrp_1	KSMBR1_2628	Q1PX08	kustc1010
	nrp_2	KSMBR1_2631	A0A2C9CHN5	kustc1014
	nrp_3	KSMBR1_3717	A0A2C9CKI9	Not identified
	nrp_4	KSMBR1_3718	A0A2C9CKK6	Not identified
NO_2_^–^/formate	focA_5	KSMBR1_3008	A0A2C9CL14	kusta0004
	Nar1 (focA_6)	KSMBR1_3009	Q1Q7F4	kusta0009
	focA_3	KSMBR1_1474	Q1PZF6	kustd1720
	focA_4	KSMBR1_1475	Q1PZF5	kustd1721
	focA_2	KSMBR1_1070	Q1Q1C7	kuste3055
	focA_1	KSMBR1_0300	Q1Q4Z9	kuste4324
	nirC	KSMBR1_0451	A0A2C9CBC5	
	HPP	KSMBR1_2720	Q1Q0U0	Kuste2872
	HPP	KSMBR1_1075	Q1Q1D1	Kuste3051
NO_3_^–^	narK_2	KSMBR1_3317	Q1Q683	kuste2335
	narK_1	KSMBR1_3299	A0A2C9CJD2	kuste2308

*Frank et al., 2018; **Strous et al., 2006, ***Speth et al., 2012.

The reason for the high number of homologous nutrient transporters in anammox bacteria is not yet known. We hypothesized that distinct transporter proteins are expressed under different growth conditions, i.e., the availability of the substrates ammonium and nitrite. Transporters with higher affinity would be upregulated upon low or limiting nutrient conditions. Nearly all previous transcriptome work in *K. stuttgartiensis* and in other anammox species has been performed under nitrite limitation with an excess of ammonium to prevent nitrite toxicity (de Almeida et al., 2011; Hu et al., 2013, 2019; Kartal et al., 2013; Neumann et al., 2014). The past body of work is therefore not fit to determine the influence of different nutrient limitations on the expression levels of transporters. In this paper, we tested the effect of ammonium- versus nitrite limitation on the expression level of the various genes encoding for nutrient transporters. Based on the results we discuss the redundancy in nutrient transport proteins in anammox bacterium *K. stuttgartiensis* MBR1.

## Materials and Methods

### Bacterial Growth

*Kuenenia stuttgartiensis* MBR1 was cultivated in a single cell membrane bioreactor (MBR) Applikon B.V. Schiedam, Netherlands) as described previously (Kartal et al., 2011a). The growth medium consisted of (per litre): 1.25 g KHCO_3_, 0.025 g NaH_2_PO_4_, 0.5 ml 1.2 M HCl, 0.15 g CaCl_2_⋅2H_2_O, 0.1 g MgSO_4_⋅7H_2_O and 6.25 mg FeSO_4_⋅7 H_2_O. The reactor volume was 2 L. The pH was maintained at 7.3 using 100 g/L KHCO_3_ buffer solution. To ensure an anaerobic system both the reactor and the main medium bottle were flushed with a mixture of Argon/CO_2_ (95%/5%) at 10 ml/min. Excess biomass was removed at 0.138 L per day, resulting in a dilution rate of 0.07 (doubling time of 10 days). For nitrite limited conditions, the reactor received 15 mmol/day NaNO_2_ and 7.5 mmol/day (NH_4_)_2_SO_4_, resulting in 15 mmol/day NO_2_^–^ and 15 mmol/day NH_4_^+^. For ammonium limited conditions, the NaNO_2_ load of the reactor was increased in two steps to 18 and finally to 21 mmol/day NaNO_2_, without changing the (NH_4_)_2_SO_4_ load. A secondary medium bottle containing all medium salts as described above but without NaNO_2_, only 5 mM (NH_4_)_2_SO_4_ and with reduced FeSO_4_⋅7 H_2_O (3.13 mg/L) to prevent precipitation, was used to add approximately 0.45 mmol (NH_4_)_2_SO_4_ per day. This allowed for fine tuning of the ammonium:nitrite ratio and enabled the nitrite levels to be kept below toxic levels, but between 2 and 20 mg/L (40 and 400 μM).

### Ammonium and Nitrite Determination

Nitrite and nitrate concentrations in the reactor were determined daily using MQuant^TM^ 10–500 mg/l nitrate detector strips (Merck KGaA, Darmstadt, Germany) during the operation of the bioreactor. Supernatants of reactor samples were used for accurate determination of nitrite and ammonium concentrations in the reactor. Nitrite concentrations were determined using the colorimetric Griess-reaction (Feigl and Amaral, 1958): 5 μl supernatant was mixed with 45 μl milliQ, 50 μl 1% sulfanilic acid in 1 M HCL and 50 μl 0.1% naphtylethylene diaminedihydrochloride in water. The solution was then incubated for 10 min at room temperature, and the absorbance was analyzed at a wavelength of 540 nm. MilliQ Water was used as a blank control.

Ammonium concentrations were determined using ortho-phtaldialdehyde (OPA), either by the colorimetric assay or the more sensitive fluorescence method (Taylor et al., 1974). For the colorimetric ammonium assay, 50 μl supernatant was mixed with 750 μl OPA reagent [0.54% (w/v) ortho-phthaldialdehyde, 0.05% (v/v) β-mercaptoethanol, and 10% (v/v) ethanol in 400 mM potassium phosphate buffer (pH 7.3)], incubated for 20 min at room temperature and absorbance at 420 nm was read using a SPEKTRAmax^TM^ plate reader (Molecular Devices, San Jose, California).

For the sensitive ammonium assay, 10 or 20 μl supernatant was mixed with 200 μl OPA reagent that had been diluted 10-times in 400 mM potassium phosphate buffer (pH 7.3). Samples were incubated in the dark for 20 min and fluorescence was measured using a Spark^TM^ plate reader (Biocompare, South San Francisco, California). Samples were shaken 5 s pre-measurement, with excitation and emission wavelengths at 411 and 482 nm, respectively.

### Sampling for Transcriptome Analysis

When the bioreactor had been growing in steady state for 8–25 days in either ammonium- or nitrite limited conditions, 2 ml samples were taken on three separate days and immediately centrifuged at room temperature for 2 min at 16,100 × *g*. The supernatant was removed, and the pellets snap-frozen in liquid nitrogen and stored at −80°C.

### RNA Isolation, Library Preparation, and Sequencing

From each of the 6 samples, total RNA was isolated using TRIzol^TM^ Reagent (Thermo Fisher Scientific, Waltham, United States) according to the manufacturer’s instructions with the exception that 100-fold higher cell numbers were used, since the recommended cell number resulted in very low to no RNA yields. The isolated RNA was treated with DNAse 1, as described and provided by the RiboPure^TM^ kit (Thermo Fisher Scientific, Waltham, United States). RNA was eluted in DEPC treated water in volumes between 20 and 25 μl of which 1–2 μl was used to quantify the isolated RNA. Concentrations ranged between 40 and 112 ng/μl and RNA Integrity Numbers (RIN) were between 8.9 and 9.4. Ribosomal RNA was partly removed using the MICROBExpress kit (Thermo Fisher Scientific) according to the manufacturer’s instructions. Although this kit removes most of the 23S rRNA, it does not remove the 16S rRNa of *K. stuttgartiensis* efficiently. RNA was fragmented to 260 bp fragments, cDNA was synthesized, adapters (including barcodes) were ligated and cDNA was amplified using the TruSeq stranded mRNA Library Prep Kit v2, according to the protocol supplied by the manufacturer (Illumina, San Diego, CA, United States). The 6 barcoded cDNA libraries were pooled prior to sequencing, which was performed with the Illumina MiSeq^®^ sequencer (Illumina, San Diego, CA, United States) using the 150 bp sequence chemistry and the Miseq Reagent Kit v3 (Illumina, San Diego, CA, United States).

### RNA and cDNA Quantification

RNA and cDNA were quantified using the Qubit^TM^ RNA HS Assay Kit and dsDNA HS Assay Kit (Thermo Fisher Scientific, Waltham, MA, United States), respectively. The quality and size distribution of the RNA before and after rRNA removal, and of the cDNA libraries was determined using the Agilent 2100 Bioanalyser with the Agilent RNA 6000 Nano Kit and the high sensitivity DNA kit (Agilent technologies, Santa Clara, CA, United States), respectively. The cDNA fragments in the libraries peaked around the recommended 260 bp.

### Sequence Reads Analysis

The number of sequencing reads per sample varied between 1.4 and 4.8 million with an average read length of 137 bp. CLC Genomics Workbench 11.0.1 (Qiagen bioinformatics, Denmark) was used to trim reads using the following settings: quality limit was set to 0.01, with a maximum of 2 ambiguous nucleotides, no adapter trimming, and removal of 10 nucleotides at the 5′ and 5 nucleotides at the 3′ end. Reads smaller than 50 bp were discarded. The number of reads left after trimming varied between 1.3 and 4.5 million with an average read length of 118 bp. Non-mRNA sequences were removed by mapping the reads to all non-coding RNAs obtained from the *K. stuttgartiensis* MBR1 genome (Frank et al., 2018). The non-mapped reads, consisting of the mRNA reads, were subsequently mapped to the annotated *K. stuttgartiensis* MBR1 genome and mapping results were expressed in RPKM values (Reads Per Kilobase gene per Million mapped reads). Average RPKM values were calculated for the triplicate samples per condition. For differential expression analysis a Wald test was performed using DEseq2 in R and adjusted p-values were calculated, taking into account the false discovery rate (Love et al., 2014). Genes with significant changes in expression between ammonium and nitrite limited growth conditions were selected using the following three criteria: (1) mean of normalized counts (basemean values) > 4, (2) log2foldchange > 0.58 or < -0.58 (i.e., a difference in expression of more than 1.5-fold), and (3) adjusted *p* < 0.05. Assignment of *K. stuttgartiensis* MBR1 genes to Clusters of orthologous groups (COG) was performed at NCBI using the Conserved Domain Search Service (Marchler-Bauer and Bryant, 2004).

### Nucleotide Sequence Accession Number

The transcriptome sequences were deposited at NCBI in the Gene Expression Omnibus (GEO) under accession number GSE148825.

## Results and Discussion

### Nitrite and Ammonium Limited Growth of *K. stuttgartiensis* MBR1

The expression of the multiple ammonium, nitrite and nitrate transporters encoded in the genome of *K. stuttgartiensis* MBR1 was studied under ammonium- and nitrite limited growth conditions. Hereto, we set up and optimized *K. stuttgartiensis* cultivation in a dedicated bioreactor system. The experiment was started with cultivation of planktonic *K. stuttgartiensis* cells in a membrane reactor with 15 mM nitrite and ammonium, resulting in nitrite limited conditions, a steady state cell density of OD_600_ 0.6 and a doubling time of 10 days. To obtain ammonium limited conditions, we increased the nitrite concentration in the medium over 2 days in two steps, via 18–21 mM nitrite, while maintaining the doubling time at 10 days and maintaining the cell density at OD_600_ 0.6. Reaching a stable steady state under ammonium limited conditions is more challenging, as the excess nitrite left in the medium can cause cell stress and toxicity and the onset of biofilm formation. We found that reducing cell densities in the reactor helped to avoid biofilm formation. Therefore, for this experiment we cultivated *K. stuttgartiensis* at a lower cell density than we usually do in our maintenance bioreactor that is run at OD_600_ 1.2. To avoid toxicity and biofilm formation, the reactor was carefully monitored for nitrite build up. A separate medium, containing 5 mM ammonium but no nitrite, was pumped into the reactor to maintain the concentration of nitrite in the reactor between 40 and 400 μM while keeping the ammonium concentration at 0 mM. Despite careful fine-tuning, it proved difficult to maintain the cell density at a steady state for more than 1 month. Several times the reactor lost biomass with concomitant increase in ciliates in the reactor, presumably consuming the *K. stuttgartiensis* cells. Also, the cells in the reactor occasionally still started producing biofilm resulting in loss of optical density.

### Transcriptomes of *K. stuttgartiensis* MBR1 Under Nitrite and Ammonium Limited Conditions

**FIGURE 1:**
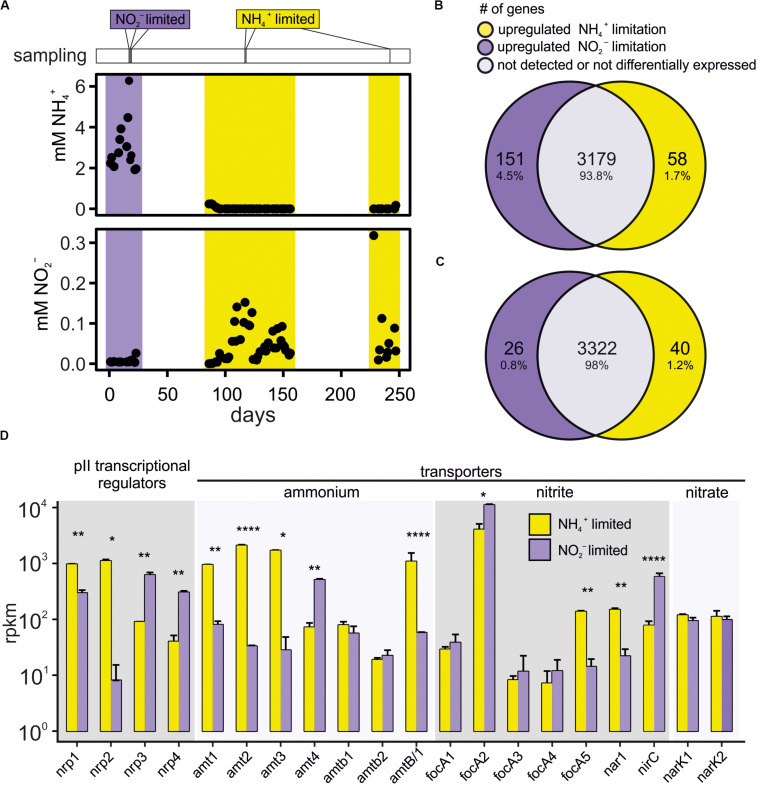
**(A)** Measured ammonium and nitrite levels in the *K. stuttgartiensis* MBR1 bioreactor and sampling times for transcriptomic analyses, indicated with vertical lines above the graph. **(B)** Venn diagram showing the number of genes that were exclusively expressed during either ammonium- (yellow) or nitrite-limitation (purple), and the genes that were expressed in both conditions. **(C)** Venn diagram showing only the genes significantly upregulated > 1.5-fold in either ammonium- (yellow) or nitrite-limitation (purple). The overlap includes all genes with either (1) very low expression levels (basemean < 4), or (2) expression levels that were within 1.5-fold change between ammonium- and nitrite limited conditions, or (3) with changes in expression levels that were below the significance level (padj > 0.05). Note that the 707 genes not expressed in either condition are not included in the Venn diagrams. **(D)** Bar graph of P_*II*_ regulator genes (nrp1-4), ammonium transporter genes (amt1-4, amtb1-2, and amtB/1), nitrite transporter genes (focA1-5 and nar1), nitrate transporter genes (narK1-2) and their expression levels under both ammonium- (yellow) and nitrite (purple) limited growth. Statistical significance ns: *p = 0.05, **p = 0.01, ****p = 0.0001. RPKM values corresponding to each bar are shown in Supplementary File 2.

We used transcriptomics to investigate and compare differential gene expression under the two growth conditions. Gene expression of cells grown under nitrite- versus ammonium limited continuous culture conditions was compared using triplicate samples for each condition (Figure 1A). Before analysis the reads mapping to 53 structural RNA (rRNA, sRNA, and tRNA) genes were removed from the data set. Of the total of 4095 (minus the 53 structural RNA genes) annotated *K. stuttgartiensis* MBR1 genes, transcripts of 654 genes were not detected in either ammonium or in nitrite limited growth conditions. Over half of these genes (474) were annotated as hypothetical protein or putative ORF and 36 genes were either insertion elements or annotated as (putative) transposases. Of the genes that were expressed, 3179 were expressed in both growth conditions; 58 genes were only detected during ammonium limited growth; and 151 were only detected during nitrite limited growth (Figure 1B). The 58 genes that were expressed only during ammonium limitation showed very low to medium expression levels (average 12 and a maximum expression of 58 RPKM). Only seven of these genes were annotated, of which two genes involved in flagellum biosynthesis, a gene containing a DNA polymerase 3 finger domain, and rubredoxin gene *rub_1*, had expression levels above 4 RPKM (Supplementary File 1). The 151 genes that were expressed only during nitrite limited conditions also had low to medium expression levels (average 12 and a maximum of 79 RPKM). Only 23 of these genes were annotated and 18 had an expression of > 4 RPKM or more (Supplementary File 1). These included four transposon-like elements, a flagellar biosynthesis gene, and rubredoxin gene *rub_2*. Rubredoxins are iron containing electron carriers. They were recently found to be expressed in *Brocadia sinica* when grown using an electrode instead of nitrite as terminal electron acceptor and they were proposed to shuttle electrons from the anammoxosome to the exterior (Shaw et al., 2020). *K. stuttgartiensis* encodes three rubredoxin genes and the third gene, KSMBR1_2919, is expressed at around 300 RPKM in both nitrite and ammonium limited conditions so this gene may encode the dominant rubredoxin present in the cell. It is unclear at present what the role of the rubredoxins is in ammonium- and nitrite-limited growth conditions.

Of the genes that were expressed in both conditions, we found 40 genes significantly upregulated during ammonium limited growth versus 26 genes during nitrite limited growth (Figure 1C and Supplementary File 2). When assigned to categories of orthologous groups, these genes mainly cluster in C (energy production and conversion), P (inorganic metabolism, including the nutrient transport genes) and T (signal transduction) (Supplementary Figure 1). Most genes (2950) were not assigned to any orthologous group, similar to reports for other anammox species (van de Vossenberg et al., 2013; Bagchi et al., 2016).

**FIGURE 2:**
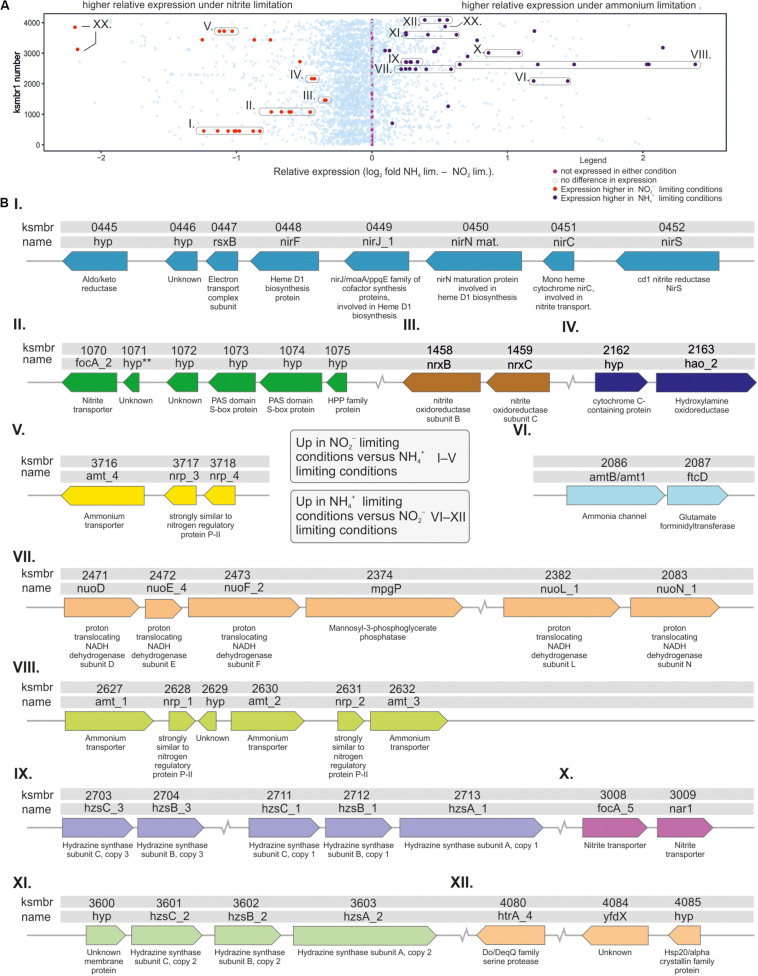
Schematic representation of *K. stuttgartiensis* MBR1 genes that are either up- or downregulated in response to ammonium- or nitrite-limiting bioreactor growth conditions. **(A)** graph showing all genes as dots on vertical line, with the relative expression on the y-axis. Genes indicated in translucent blue were either not significantly expressed, or not significantly different between the two conditions. Genes indicated in purple were expressed at a significantly higher level in response to ammonium limitation compared to nitrite limitation. Genes indicated as red dots were expressed at a significantly higher level in response to nitrite limitation when compared to ammonium limitation. Circles were drawn around genes that are in very close genomic proximity. XX = KSMBR1_3177, KSMBR1_3122, and KSMBR1_3846. **(B)** Synteny and description of genes that were expressed at a significantly different level between nitrite- and ammonium-limiting conditions. Roman numerals I–V indicate genes that were expressed relatively more during nitrite-limiting conditions, and roman numerals VI–XII indicate genes expressed relatively more during ammonium-limiting conditions.

Many of the differentially expressed genes were organized in operon-like structures (Figure 2). Of the putative operons that were significantly upregulated in nitrite limited conditions, one contained nitrite transporter gene *focA_2* and another ammonium transporter gene *Amt_4* (Figures 2BII,V) and three code for enzymes involved in nitrite metabolism (Figures 2BI,III,IV). Putative operons that were significantly upregulated in ammonium limited conditions, contained nutrient transporter genes (Figures 2BVI,VIII,X), one contained *nuo* genes that encode NADH dehydrogenase subunits (Figure 2BVII, see also below), and two contained in total all three copies of the hydrazine synthase cluster (Figures 2BIX,XI, see also below). A seventh putative operon contained a gene encoding Hsp20/alpha crystallin family protein, a Yfdx protein with unknown function and a Do/DeqQ family serine endopeptidase.

### Ammonium Transporters

Of all seven *K. stuttgartiensis* MBR1 ammonium transporters the *amt_*1 (KSMBR1_2627), *amt_*2 (KSMBR1_2630) and *amt_*3 (KSMBR1_2632) cluster (Figure 2BVIII), and *amtB*/*amt*1 (KSMBR1_2086, Figure 2BVI) were significantly upregulated in response to ammonium limitation. The *amtB/amt1* gene has an additional N-terminal concanavalin A-like domain as found in β-xylosidases (Jordan and Wagschal, 2010), that is transcribed with the amtB part of the gene as one transcript (data not shown). Concanavalin A is a lectin, but it is unclear at present whether its role in the AmtB/amt1 protein is indeed carbohydrate binding. The *amtB/amt1* homolog was previously identified as one of the rare prokaryotic Rh members of the AmtB/Mep/Rh transporter family. In the ammonia oxidizer *Nitrosomonas europaea*, which lacks AmtB transporters, this Rh homolog is used to transport ammonium into the cell (Lupo et al., 2007; Weidinger et al., 2007; Matassi, 2017). However, the function of the AmtB/amt1 in *K. stuttgartiensis* remains to be elucidated.

In response to nitrite limitation the ammonium transporter *amt_4* (KSMBR1_3716, Figure 2BV) was significantly upregulated (Figure 1D), reflecting results obtained for ammonium limited *Scalindua brodae* (Russ et al., 2019). The two ammonium transporters with histidine kinase domains, *amtb_*1 (KSMBR1_3722) and *amtb_*2 (KSMBR1_3866), were expressed at a low level (<100 RPKM) and expression did not differ between the tested conditions (Figure 1D). The *amtb*2 gene product (named KsAmt-5 by the authors) has been shown to function as an ammonium sensor *in vivo*, but does not transport ammonium across the membrane (Pfluger et al., 2018). In fungal species, multiple Mep-type ammonium transporters are present as well and some of these ammonium “transceptors” have a signaling function (van den Berg et al., 2019). So it is likely that in *K. stuttgartiensis* more than one ammonium transporter homologs has a function other than transport.

GlnK P_*II*_ regulators regulate ammonium transport by binding near the exit port and thereby blocking the transport channel under high ammonium conditions (Conroy et al., 2007; Gruswitz et al., 2007). These GlnK P_*II*_ regulators and AmtB transporters are well known to be conserved gene pairs in prokaryotes (Thomas et al., 2000; Coutts et al., 2002). Four GlnK P_*II*_ regulators are present in the genome of *K. stuttgartiensis* MBR1. Regulators *nrp1* (KSMBR1_2628) and *nrp2* (KSMBR1_2631) were upregulated in response to ammonium limitation (Figure 1D), and are located next to *amt_1*, *amt_2*, and *amt_3*. These genes likely form one operon as the transcriptome reads overlap in the whole region between *amt_1* and *amt_3* (Figure 2BVIII and data not shown). Regulators *nrp*3 (KSMBR1_3717) and *nrp*4 (KSMBR1_3718) were upregulated in response to nitrite limitation (Figure 1D) and overlapping transcriptome reads indicated these form an operon with *amt_4* (Figure 2BV and data not shown).

Although anammox bacteria contain a strikingly large number of ammonium transporters, the presence of more than one *amtB* copy in one organism is not unusual. In fact, although model organism *Escherichia coli* only has one *amtB* gene this appears to be an exception, as multiple homologs of *amtB* genes and of the other members in the AMT/MEP/Rh ammonium transporter family have been observed in organisms from all three domains of life (Merrick et al., 2006; Andrade and Einsle, 2007; Tremblay and Hallenbeck, 2009; McDonald and Ward, 2016). In plants and yeast with multiple transporters, some have been identified to differ in their affinity to ammonium (Loqué and von Wirén, 2004; Qin et al., 2018). Our results indicate that this might also be the case in *K. stuttgartiensis*, with *amt_4* representing a low affinity ammonium transporter expressed when ammonium concentrations are relatively high, and *amt_1*, 2, 3 and *amtB/amt1* potential high affinity transporters. However, the *amtB/amt1* with the concanavalin A-like domain may have a function other than ammonium transport. It is most homologous to the Rh members of the AMT/MEP/Rh transporter family, which have rather low affinity for ammonium (Lupo et al., 2007; Baday et al., 2015) and have also been associated with CO_2_ transport (Peng and Huang, 2006).

In addition to the observed differential expression of potential high and low affinity transporters, and the indication that some of the transporters may have alternative substrates, it is also possible that different transporters are located in the different membranes of the anammox cell. The anammox reaction takes place in the anammoxosome meaning that both substrates ammonium and nitrite and by-product nitrate need to cross three double membranes: the outer-, cytoplasmic- and anammoxosome membranes. The three membranes could each have separate dedicated transporters. Indeed, Medema et al. (2010) predicted Amt_1 to be located in the cytoplasmic membrane and Amt_2 and -3 in the anammoxosome membrane. At present it is unclear how membrane proteins would be specifically targeted to the different membranes, as signal sequences have not been identified. It has therefore been suggested that chaperones may be required (Medema et al., 2010). Location of Amt transporters on the anammoxosome membrane poses the intriguing question of how these transporters could be regulated by their P_*II*_ regulators. These bind at the exit of the Amt pore in the cytoplasm, blocking ammonium transport (Conroy et al., 2007; Gruswitz et al., 2007). The method of regulation has been well studied for ammonium uptake in assimilatory processes by the GS/GOGAT cycle in *E. coli*: a drop in internal ammonium concentration results in accumulation of 2-oxoglutarate in the cytoplasm, which binds to GlnK (the P_*II*_ regulator) together with UDP and ATP. This causes release of GlnK from AmtB and subsequent transport of ammonium into the cell (Kim et al., 2012; Huergo et al., 2013). Regulation of Amt transporters in the anammoxosome membrane of *K. stuttgartiensis* by the same mechanism would require the GS/GOGAT system as well as a large part of the central carbon metabolism present inside the anammoxosome, which is unlikely as biosynthetic processes most probably take place in the cytoplasm. Whether ammonium import is differently regulated when ammonium is used for dissimilation rather than only for assimilatory processes is not known. A parallel with aerobic ammonium oxidizers cannot be drawn here as these organisms are thought to oxidize ammonium in the periplasm and they do therefore not have to import ammonium for dissimilation into the cell (Lehtovirta-Morley, 2018). Not all Amt transporters appear to be regulated through a P_*II*_ regulator (Strösser et al., 2004; Tremblay et al., 2007), and this could be the case for potential Amt transporters in the anammoxosome membrane as well. Alternatively, it is possible that in anammox cells, high affinity ammonium transporters located on the cytoplasmic membrane concentrate ammonium/ammonia in the cytoplasm enough to allow sufficient diffusion of ammonia into the anammoxosome without additional dedicated transporters, or with help of a low affinity transporter. Since hydrazine can diffuse freely through dense ladderane membranes that are characteristic for anammox cells (Moss et al., 2018), the smaller ammonia will also be able to diffuse through, with or without a facilitator.

### Nitrite and Nitrate Transporters

The genome of *K. stuttgartiensis* MBR1 encodes for six nitrite *focA* transporters. Multiple copies of nitrite transporters in one organism have been described for other organisms, but they are not usually all of the *focA* type (Clegg et al., 2002). Of the 6 *focA* transporters, *focA_2* (KSMBR1_1070, Figure 2BII) was highly expressed in both conditions and further upregulated in response to nitrite limitation, and *focA_5* (KSMBR1_3008) had low expression but was upregulated under ammonium limiting conditions (Figure 1D). The transporters *focA_1* (KSMBR1_0300), *focA_3* (KSMBR1_1474) and *focA_4* (KSMBR1_1475) were barely expressed under either condition (∼38, 12, and 12 RPKM, respectively). In addition to the five annotated *focA* genes, a sixth *focA* homolog is annotated as the nitrate oxidoreductase *nar*1 (KSMBR1_3009, Table 1). This gene is located adjacent to *focA_5* (Figure 2BX) and is upregulated (from 22 to 127 RPKM) upon ammonium limitation. Although PROKKA annotates it a *nar*1, BlastP analyses and information reported in the genome browser MaGe (Vallenet et al., 2013) suggest that this transporter belongs to the formate/nitrite transporter family instead. Previously, the homologous gene in strain *K. stuttgartiensis* RU1 was manually annotated as a *focA* formate/nitrite transporter (kusta0009) (Strous et al., 2006). We therefore conclude that *nar*1 is a nitrite transporter and suggest renaming it *focA_6* (Table 1). We identified a further nitrite transporter homolog, *nirC* (KSMBR1_0451) that showed upregulation (from 83 to 623 RPKM) during nitrite limited growth.

The hypothetical genes KSMBR1_2720 (Supplementary Table 1) and KSMBR1_1075 (Figure 2BII) were upregulated in response to nitrite limitation and are predicted to be part of the HPP protein family. This recently described protein family consists of membrane proteins that have four trans-membrane domains and a conserved HPP motif. In cyanobacteria and chloroplasts, a HPP protein was shown to play a role in nitrite transport (Maeda et al., 2014). In addition, KSMBR_1075 is located in close proximity to the most highly expressed nitrite transporter *focA_2*. This suggests that KSMBR1_2720 and KSMBR_1075 may also function as nitrite transporters in *K. stuttgartiensis* and brings the total nitrite transporter homologs in the genome to nine.

In a study where *K. stuttgartiensis* RU1 was cultivated on nitric oxide and ammonium (without nitrite), the expression of the *focA_2* homolog kuste3055 and the *nirC* homolog kuste4137 was downregulated 36- and 27-fold respectively (Hu et al., 2019). FocA/NirC transporters are relatively non-selective and can also transport other small anions (Lu et al., 2012; Lu et al., 2013). Therefore it is possible that FocA_2 and perhaps also NirC constitute the main nitrite transporters and the other FocA homologs may have a physiological function other than nitrite transport in *K. stuttgartiensis*. As anammox bacteria are able to use formate as an alternative electron donor to ammonium (Kartal et al., 2007, 2008; van de Vossenberg et al., 2008), transport of formate is a likely function for one or more of the other FocA transporters.

The genome also encodes for two nitrate/nitrite transporters, both of the NarK2-type, named *narK*1 (KSMBR1_3299), and *narK*2 (KSMBR1_3317), which were constitutively expressed at around 100 RPKM and did not respond to the investigated nutrient limitations. The NarK transporters are thought to export the by-product nitrate, which is produced from nitrite, probably by the nitrite oxidoreductase Nxr complex, thereby supplementing the electrons lost from the electron transport chain to carbon fixation (Reimann et al., 2015; Kartal and Keltjens, 2016). As the growth rates in both the ammonium- and nitrite limited conditions was kept the same, the rate of electrons lost to carbon fixation would be similar in both conditions and therefore the rate of nitrate production by Nxr is not expected to vary significantly between the two conditions.

### Genes Involved in Energy Metabolism

**FIGURE 3:**
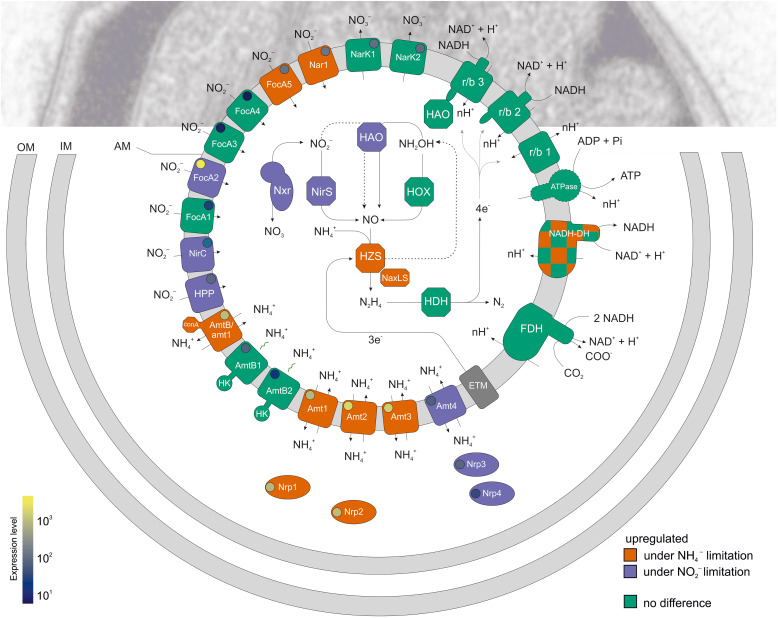
A schematic overview of a *K. stuttgartiensis* cell and the differential expression of gene products related to metabolism and nutrient transport under nitrite- versus ammonium limited bioreactor growth conditions. Transporters have been drawn on the anammoxosome membrane for comparison but their localization on either of the three anammox membranes awaits experimental validation. Genes up-regulated under nitrite- relative to ammonium limiting conditions are indicated in purple and genes upregulated under ammonium- relative to nitrite-limiting conditions are indicated in orange. Genes that are not affected when grown under either nitrite- or ammonium-limiting conditions are indicated in green. A circle within each transport related gene indicates relative expression level. OM, outer membrane; IM, inner (cytoplasmic) membrane; AM, anammoxosome membrane; HK, histidine kinase domain. Proteins depicted in this image: **Ammonium transporters:** AmtB/amt1 (KSMBR1_2086), Amt_1 (KSMBR1_2627), Amt_2 (KSMBR1_2630), Amt_3 (KSMBR1_2632), Amtb_2 (KSMBR1_3866), Amtb_1 (KSMBR1_3722), Amt_4 (KSMBR1_3716); **Ammonium transport P_*II*_ regulators**: Nrp1 (KSMBR1_2628), Nrp2 (KSMBR1_2631), Nrp3 (KSMBR1_3717), Nrp4 (KSMBR1_3718); **Nitrite transporters:** FocA_5 (KSMBR1_3008), Nar1 (focA_6) (KSMBR1_3009), FocA_3 (KSMBR1_1474), FocA_4 (KSMBR1_1475), FocA_2 (KSMBR1_1070), FocA_1 (KSMBR1_0300), HPP (Putative nitrite transporter, KSMBR_1075); **Nitrate transporters:** NarK1 (KSMBR1_3317), NarK2 (KSMBR1_3299); **Anammox key metabolism:** NirS (KSMBR1_0452), HZS (*hzsA*: KSMBR1_2705, 2713, 3603), *hzsB*: (KSMBR1_2704, 2712 and 3602), and *hzsC*: (KSMBR1_2703, _2711, and 3601), NXR (A: KSMBR1_1455, B: 1458, and C: 1459), HAO (KSMBR1_2163), HAO r/b3 (KSMBR1_3792), HOX (KSMBR1_2670); **Electron transport chain:** FDH (KSMBR1_2469, 2470, 2471, 2479), r/b 1 (KSMBR1_1020 – 1021), r/b 3 (KSMBR1_ 3792 – 3797), r/b 2 (KSMBR1_1275 – 1283), ATPase (KSMBR1_0120 – 0130), NADH-DH (KSMBR1_3978 – 3990), ETM (unknown electron transport module).

Several genes whose products are involved in the anammox reaction were differentially regulated in ammonium- versus nitrite limited conditions (Figures 2B, 3). The genes significantly upregulated during nitrite limitation included those whose gene products require nitrite as a substrate: (1) the *nirS* gene (KSMBR1_0452) encoding a nitrite reductase, a candidate for catalyzing the conversion of nitrite to nitric oxide in the anammox reaction (Kartal et al., 2013). This gene was expressed at a low level (100 RPKM), but was 10-fold upregulated in response to nitrite limitation. The *nirS* gene is situated adjacent to the *nirC* nitrite transporter homolog (KSMBR1_0451) described above. (2) The nitrite oxidoreductase genes *nrxCB* (Figure 2BIII). Please note there is a typing error in the text of the KSMBR1 annotation here: *nrx* instead of *nxr*. These are the homologs of the *nxrCB* genes kustd1704 and 1703 in *K. stuttgartiensis* RU1 (Strous et al., 2006; Kartal and Keltjens, 2016) and are part of a larger gene cluster that encodes the nitrite oxidoreductase complex Nxr (see above). The Nxr complex in *K. stuttgartiensis* consists of a catalytic α subunit (NxrA), a β subunit thought to function as an electron channel (NxrB) and a γ subunit (NxrC) that has a heme b binding domain and is homologous to NxrC subunits from other organisms that function as reductases or dehydrogenases (Reimann et al., 2015). All *nxr* subunits (A: KSMBR1_1455, B: 1458, and C: 1459), were highly expressed and subunit B and C were upregulated in response to nitrite limitation. (3) The hydroxylamine oxidoreductase HAO_2 (KSMBR1_2163), which is one of ten paralogous HAO type genes present in *K. stuttgartiensis* MBR1, was expressed at a high level (2000 RPKM), and was also 2.5-fold upregulated in response to nitrite limitation.

The upregulation of the three genes *nxrBC*, *nirS*, and *hao2* when growth was limited by nitrite may indicate that the affinities of the enzymes are too low to sustain enzyme activity and maintain the growth rate as the level of nitrite provided drops. This low affinity allows the organism to regulate the metabolic flux by modifying the amount of enzyme in the cell. Interestingly, both *nirS* and *hao2* were upregulated. Both these enzymes have been proposed to be the nitrite reductase responsible for the conversion of nitrite to nitric oxide: In *K. stuttgartiensis* RU1, the *nirS* gene is expressed at lower levels compared to other genes involved in the energy metabolism and therefore the hydroxylamine oxidoreductase HAO_2 homolog kustc0458 is hypothesized to encode the enzyme that reduces nitrite to nitric oxide instead (Kartal et al., 2013). Our results show that both are expressed and upregulated under nitrite limitation, with *hao2* showing 5-fold higher expression than *nirS*. Interestingly, Hu et al. (2019) reported that in *K. stuttgartiensis* RU1 cells cultivated with nitric oxide and ammonium rather than nitrite and ammonium, the nitrite reductase gene cluster (containing *nirS* and *nirC*), as well as the *hao2* homolog kustc0458 were amongst the most *downregulated* genes in the transcriptome (Hu et al., 2019). So although *nirS* and *hao2* are upregulated when nitrite concentrations are low, they shut down when nitrite is absent. This suggests that the expression of *nirS* and *hao2* is regulated by a mechanism that does not respond linearly to the concentration of nitrite. It remains unclear whether one or both of these genes encode(s) the physiological nitrite reductase that produces the nitric oxide used by HZS – an important topic for future research.

In the next step in the anammox reaction, nitric oxide and ammonium are converted to hydrazine by the unique HZS complex, which consists of three subunits and two copies of each subunit (Dietl et al., 2015). In the genome of *K. stuttgartiensis* MBR1, there are three copies of the *hzs* operon present, all of which were very highly expressed. For comparison, *hzsC_1* (KSMBR1_2711) is expressed at 12475 RPKM and *hzsA_1* (KSMBR1_2713) at 28462 RPKM compared to genes involved in carbon fixation formate dehydrogenase which is expressed at 1584 RPKM and CO dehydrogenase (KSMBR1_3598)/acetyl-CoA synthase at 652 RPKM under nitrite-limited conditions. All copies of both *hzsB* (KSMBR1_3601, 2704, and 2712) and *hzsC* (KSMBR1_2703, 2711, and 3601) were upregulated in response to ammonium limitation, while only one copy of *hzsA (hzsA*_1, KSMBR1_2713) was upregulated (Figures 2BIX,XI, 3). However, as the three copies of the *hzs* cluster have nearly 100% identical gene sequences, most transcripts could map to all three copies. The algorithm maps each read only once so we could not determine from our data whether one or all *hzs* operons were highly transcribed. HZS is a slow enzyme when tested *in vitro*, but whether this is due to loss of activity during purification is not yet clear (Maalcke et al., 2014; Dietl et al., 2015). In all transcriptome studies of anammox species to date, the *hzs* genes were among the most highly expressed genes in the genome (Kartal et al., 2011b; Hu et al., 2013; van de Vossenberg et al., 2013; Bagchi et al., 2016; Russ et al., 2019). Also, in proteomics studies the HZS complex was shown to be a major part of the total protein complement (Kartal et al., 2011b; Hu et al., 2019) and maintained at these high levels even after 60 days of starvation (Bagchi et al., 2016; Ma and Wang, 2018). Even though HZS forms such a large part of total protein content of the cell, the *hzs* genes are still upregulated in response to ammonium limitation to ensure sufficient hydrazine production capacity. Russ et al. (2019) observed similar upregulation of the *hzs* genes of the marine anammox species *Scalindua brodae* during short term ammonium limitation (Russ et al., 2019). The large amount of HZS present in the cell is apparently not enough to maintain the level of hydrazine synthesis when ammonium levels are low, i.e., the affinity to ammonium is insufficient to maintain the rate of hydrazine synthesis.

Some of the genes predicted to be part of the electron transport chain were also upregulated in response to ammonium limitation, such as the complex I NADH-quinone oxidoreductase subunits D, E, F, G, L, and N (KSMBR 2471—2474, 2482, and 2485). The other subunits of this complex had higher, but not significantly higher, expression levels in ammonium- versus nitrite limited growth (Figures 2BVII, 3). The heterodimeric complex NaxLS with low redox potential (c-type cytochrome KSMBR1_3082 and KMSBR_3083) was highly expressed in both nitrite- and ammonium limited conditions (around 8,200 versus 10,000 RPKM, respectively) and the upregulation in response to ammonium limitation was significant (Supplementary Table 1). This complex binds to the HZS complex, and is hypothesized to serve one of two roles: to retain the volatile anammox intermediate nitric oxide inside the anammox cell and to shuttle it to hydrazine synthase or to shuttle electrons from an electron donor to hydrazine synthase (Ukita et al., 2010; Akram et al., 2019b). Akram et al. (2019b) hypothesize that if this complex is involved in retaining and shuttling nitric oxide, it should be upregulated in response to nitrite limitation to retain the precious nitric oxide within the cell. Indeed Hu et al. (2019) cultured *K. stuttgartiensis* on non-limiting amounts of nitric oxide instead of nitrite and observed 20- and 10-fold *down*regulation of the *naxLS* genes, respectively. However, we found *up*regulation during ammonium limitation when nitrite was not limiting, suggesting increased need when nitrite is *not* the limiting growth nutrient. As we also found upregulation of the *hzs* genes in ammonium limited conditions, it is possible that higher copy numbers of NaxLS are required to make sure all the HZS complexes in the anammoxosome are provided with sufficient NO.

### Genes Involved in Ammonium Assimilation

During ammonium limitation, the dissimilatory enzymes have to compete with the assimilatory ammonium pathways for their substrate. Ammonium assimilation can occur via the high affinity GS/GOGAT system where glutamine synthase forms glutamine from glutamate and ammonium. Glutamate synthase subsequently transfers the amide group from glutamine to 2-oxoglutate (α-ketoglurate) to produce two glutamate molecules. Alternatively, ammonium can be assimilated by the low affinity glutamate dehydrogenase (GDH) which produces glutamate from 2-oxoglutarate and ammonium (Yan, 2007). *K. stuttgartiensis* MBR1 possesses two glutamine synthase genes, *glnA_1* (KSMBR1_0034) and *glnA_2* (KSMBR1_0678), and two hypothetical proteins with homology to glutamate synthase (KSMBR1_1164 and KSMBR1_3563). It also has two copies of the low affinity ammonium assimilation GDH gene *gdh_1* (KSMBR1_1085) and *gdh_2* (KSMBR1_2236). The GS/GOGAT genes were more highly expressed than the GDH genes but none showed upregulation during ammonium limited growth (Supplementary File 2), indicating that *K. stuttgartiensis* cells were able to maintain the intracellular concentration of ammonium during ammonium limited growth conditions. This is in contrast with the marine anammox species *S. brodae*, which did induce the GS/GOGAT genes when grown under ammonium limitation (Russ et al., 2019).

### Other Genes

Three putative transposases showed significant differential expression: KSMBR_3038, an IS66 family transposase, was upregulated in response to ammonium limitation, and both KSMBR1_3122, an IS1634 family transposase, and KSMBR1_3846, an ISAs1 family transposase, were upregulated in response to nitrite limitation (Figure 2A and Supplementary Table 1).

The hypothetical gene KSMBR1_3177 was highly upregulated in response to ammonium limitation (Figure 2A). This gene has a large beta-barrel porin-2 (BBP2) domain, specific to outer membrane porins. This hypothetical porin may be upregulated to allow for a higher influx of ammonium from outside the cell. The glutamate formimidoyltransferase gene *ftcD* (KSMBR1_2087) is located just downstream of *amtB/amt1* (KSMBR1_2086) on the genome and was upregulated in response to ammonium limitation (Figure 2BVII). Glutamate formimidoyltransferases are involved in histidine degradation to glutamate. There was some but not much transcript overlap between *amtB/amt1* and *ftcD* and the expression of *ftcD* was much lower than *amtB/amt1* so we conclude the genes are probably not forming an operon.

## Conclusion

In summary, we have shown differential gene expression for several of the ammonium and nitrite transporter homologs in *K. stuttgartiensis* cells and this may partly explain the high copy number of these transporter genes in the genome. It is also clear that at least one of the ammonium transporters has a different function as an ammonium sensor rather than transporter (Pfluger et al., 2018). In addition, it is possible that specific transporters are located in different membranes in the cell. Our next step will be to investigate these other possible hypotheses for the high number of nutrient transporters in the *K. stuttgartiensis* genome. Although transcriptome analysis is a powerfool tool to study differential expression of genes, RNA levels do not necessarily reflect the levels of active protein in the cell. We therefore aim to follow up this work with proteome and activity studies to obtain more complete understanding of the physiology of *K. stuttgartiensis* in different growth conditions. The environmentally- and industrially- relevant anammox bacteria are food for thought with their compartmentalized energy metabolism. Investigating nutrient transport in a “complex” bacterial cell will ultimately help us understand how the cell regulates transport of nutrients and expression of transporters and how it responds to, and deals with, changing nutrient conditions in its environment.

## Data Availability Statement

The datasets generated for this study can be found at NCBI in the Gene Expression Omnibus (GEO) under accession number GSE148825.

## Author Contributions

MS, HC, and LN conceived parts of the project. MS and LN designed and discussed the experimental set-up. MS, DB, and GN designed, set-up, and maintained the bioreactors. MS, TA, and DB performed the experiments. MS, DB, SP, and LN analyzed the data. MS and SP prepared the figures. SP, MS, and LN wrote the manuscript with input from all authors. All authors were involved in research discussions.

## Conflict of Interest

The authors declare that the research was conducted in the absence of any commercial or financial relationships that could be construed as a potential conflict of interest.
