# Supplementary material for: Nutrient Limitation Causes Differential Expression of Transport- and Metabolism Genes in the Compartmentalized Anammox Bacterium Kuenenia stuttgartiensis
Source: Front Microbiol. 2020 Aug 13;11:1959. doi: 10.3389/fmicb.2020.01959 (PMC7438415; doi:10.3389/fmicb.2020.01959)
Supplement: Supplementary file 3 [file Image_1.PDF]

Supplementary Material

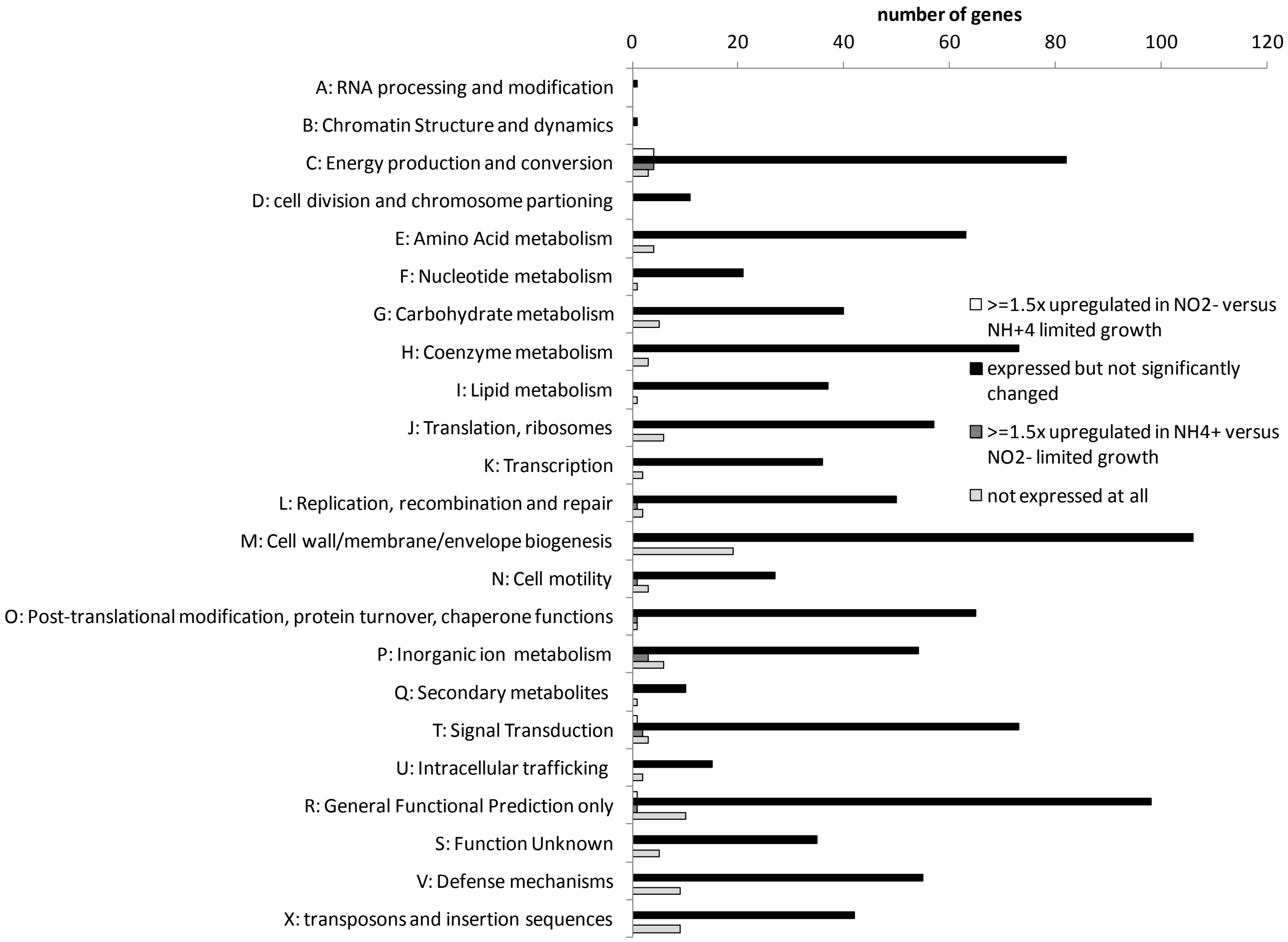

Supplementary Figure 1. Cluster of Orthologous Groups (COG)-based functional analysis of *K. stuttgartiensis* genes expressed during ammonium- and/or nitrite limited bioreactor growth conditions. White bars: number of genes significantly more than 1.5-fold upregulated in nitrite limited growth versus ammonium limited growth conditions. Black bars: number of genes with either expression changes within 1.5-fold, or with very low expression levels (basemean<4), or with changes in expression levels that were below the significance level (padj>0.05). Dark grey bars: number of genes significantly more than 1.5-fold upregulated in ammonium limited growth versus nitrite limited growth conditions. Light grey bars: number of genes not expressed in either growth condition. COG analysis was performed using [Conserved Domain Search Service (CD Search)] at NCBI. Note: 2928 out of the total 4095 *K. stuttgartiensis* genes did not have a COG function assigned to it.
